# Methylation Modification, Alternative Splicing, and Noncoding RNA Play a Role in Cancer Metastasis through Epigenetic Regulation

**DOI:** 10.1155/2021/4061525

**Published:** 2021-10-06

**Authors:** Bin Yu, Xin Yu, Jianping Xiong, Mei Ma

**Affiliations:** ^1^Department of General Surgery, The First Affiliated Hospital of Nanchang University, Nanchang, Jiangxi Province 330006, China; ^2^Department of Oncology, The First Affiliated Hospital of Nanchang University, Nanchang, Jiangxi Province 330006, China

## Abstract

Metastasis is the leading cause of cancer-related deaths. Understanding the pathogenesis of metastasis at the molecular levels is of great significance for cancer research. However, the molecular diagnosis or treatment of cancer metastasis is limited. Accumulating and growing evidence shows that epigenetic changes are present in all human cancers, and epigenetic regulation is an indispensable factor to promote tumor metastasis. With the deepening of research and the advancement of technology, the function and mechanism of epigenetic regulation, including DNA methylation, histone/RNA modification, and precursor messenger RNA alternative splicing and noncoding RNAs, has become more increasingly clear. At present, the application of epigenetic therapies in tumor treatment is becoming a feasible therapeutic route. In this review, we looked for the key molecules in epigenetic regulation and discuss their relative regulating mechanisms in cancer metastasis. Furthermore, we highlight promising therapeutic strategies, including monitoring serum DNA for diagnostic purposes and early phase clinical trial therapies that target DNA and histone methylation. This may also be beneficial in finding new targets for further prognosis and diagnosis of cancer metastasis.

## 1. Introduction

Cancer is one of the major public health problems which has become a dominant cause of death in the global world, while the stage of metastasis is responsible for approximately 90% of deaths [1]. In the early phases, uncontrolled multiplication of tumor cells leads to the formation of expanded tumor mass that affects normal tissue through physical stress. With the gradual growth of the tumor, metastasis then occurs step by step. The primary tumor cells invade the adjacent tissues, migrate to new sites, survive and adapt to the microenvironment of distant tissues, and form secondary tumors (colonization) [2, 3], which mean going to the most fearsome stage of cancer [2]. Therefore, understanding the molecular mechanism of metastasis is of great significance for the diagnosis of advanced cancer, delaying tumor progression, and finding new therapeutic targets. It is widely accepted that mutations in key genes drive cancer formation at the genetic level [4]. However, only the genetic alterations cannot explain the mechanism of cancer metastasis [5]. A massive amount of evidence suggests epigenetic changes closely related to the process of metastasis [6–8].

**Figure 1:**
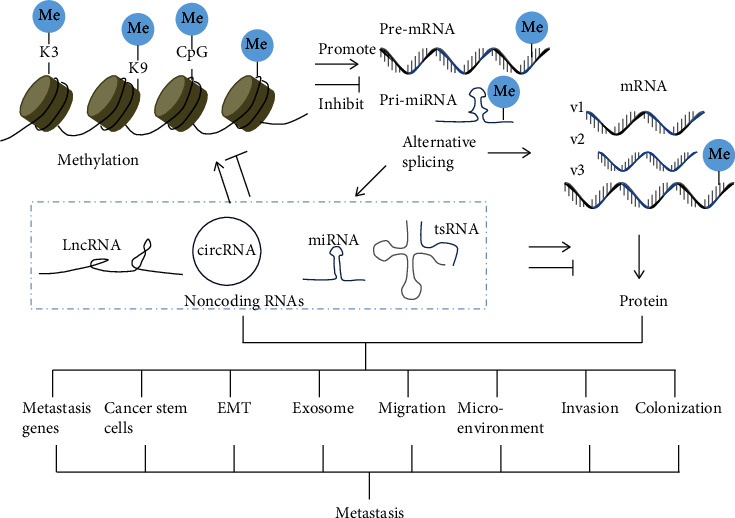
Schematic diagram of epigenetic modifications in cancer metastasis. DNA/histone methylation and RNA methylation regulate the transcription and translation of target genes, respectively. Methylation also affects alternative splicing, which increases the diversity of proteins and noncoding RNAs. Methylation, alternative splicing, and noncoding RNAs may act alone or in combination in cancer metastasis.

Epigenetic regulation refers to the heritable alterations in gene expression that are not due to the changes from the DNA sequences themselves. Since its discovery in 1983 [9], the role of epigenetic modification in cancer has become increasingly prominent with the in-depth knowledge of epigenetic mechanisms. In the past 40 years, the roles of DNA hypo- and hypermethylation in cancer have been well established, along with the reported discovery that DNA methylation at a CpG (nonmethylated cytosine/guanine base pairs) abnormality is strongly associated with cancer development [9, 10]. And in the last 30 years, imprinted genes have been found to influence cancer by chromatin modifications [11]. In the last two decades, the study of posttranscription regulation in cancer has been rapidly developed, accompanied with the application of new technologies. For example, the widespread use of next generation sequencing and the development of bioinformatics analysis have been beneficial in studying the role of different subtypes of the same gene in cancer. Here, we review the functional roles and mechanisms of epigenetic regulation in cancer metastasis (Figure 1). It may be beneficial in providing new insights into the diagnosis, prognosis, and prevention of cancer research.

### 1.1. Methylation Influences Metastasis-Associated Gene Expression

Methylation is one of the well-known modifications in DNA, RNA, and histone, which are catalyzed through methyltransferases (writers) with thorough mechanisms about how to write, read, and erase the covalent epigenetic marks [12]. Dynamic changes in methylation of DNA, RNA, and histone are essential for cellular functions. As the effect of methylation on carcinogenesis is addressed elsewhere, we pay attention to the alteration of methylation in cancer metastasis.

#### 1.1.1. DNA Methylation

5-Methylcytosine (5mC) is one of the most studied modifications of DNA methylation written by the family of DNA methyltransferase 1-3 (DNMT1-3) [13]. 5mC was erased through ten-eleven translocation (TET) dioxygenase-mediated oxidation to generate 5-hydroxymethylcytosine (5hmC), 5-formylcytosine (5fC), and 5-carboxylcytosine (5caC) [14]. In order to identify whether its function is to activate or suppress gene expression, the position of DNA methylation throughout the genome needs to be considered [15]. Whole-genome profiling of 5mC has shown that 5mC dynamically exists in enhancers, gene bodies, and extended transcriptionally inactive partially methylated domains, implicating its important function involved in disease progression, including cancer [16]. Various DNA methylation pathways in cancer metastasis have been reported and verified in tissue and cells at pancancer or specific cancers. Here, we just picked a small part of them from different cancers and cells as examples, which are listed in Table 1. Moreover, DNA modifications may serve as potential prognostic biomarkers and future therapeutic targets. For example, in breast cancer patients, serum deprivation response (SDPR) expression was silenced by promoter DNA methylation, and its expression were negatively correlated with metastasis and relapse-free survival [17]. The discovery of novel methylation of genes specifically on the metastasis of certain cancers may help reveal the underlying mechanisms and progression of specific cancer metastasis. In addition, DNA methylation could be shaped by circulating tumor cells (CTCs), which are related to the formation of metastasis seeding and promote cancer stemness and metastasis [18]. Lastly, monitoring serum DNA methylation might be used as diagnostic biomarkers to predict survival outcomes for metastatic cancers. Because aberrant DNA can be released into the bloodstream by cancer cells, it is easy to detect common tissue changes from the blood using quantitative multiplex assays, especially for cell-free DNA methylation [19]. Studies such as TBCRC 005 might provide the first potential clinical tool for measuring the serum level of methylation, which was conducive to the detection of metastatic breast cancer [20]. In another example, IRX1 promoter hypomethylation detected in serum DNA of patients with osteosarcoma might be a potential biomarker for predicting lung metastasis in osteosarcoma [21]. Compared with tissue-based biomarkers, blood DNA biomarkers were easier for repetitive monitoring and might be more informative and specific than protein biomarkers. The use of serum DNA methylation analysis might be beneficial for exploiting personalized medicine and monitoring of therapeutic effects [19].

#### 1.1.2. RNA m6A Methylation

**Figure 2:**
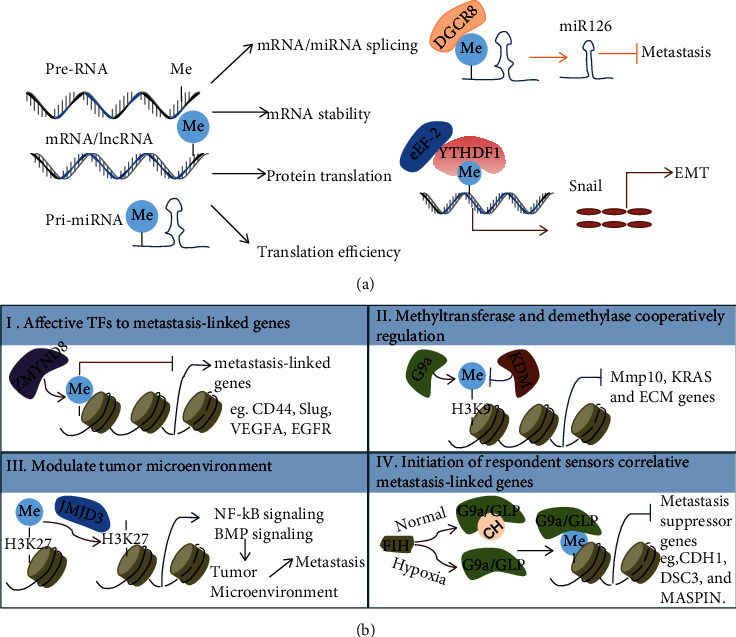
Diverse mechanisms of RNA/histone methylation in metastasis. (a) m6A methylation influenced mRNA/noncoding-RNA splicing and stabilized as well as mRNA translation. (b) Histone methylation directly or indirectly influenced the expression of target genes through transcription factors binding in their promoter areas, such as histone methylation inhibiting recruitment of the transcriptional cofactors (TFs). Histone methylation was also involved in the regulation of tumor microenvironment.

It has been more than 40 years since N6-methyladenosine (m6A) was identified [22]. m6A, one of the most abundant modifications in mRNAs, similar to DNA methylation, exists dynamically in biological effects and is influenced by “writer” (METTL3, METTL14, WTAP, and KIAA1429), “eraser” (FTO and ALKBH5), and “reader” (YTH and HNRNP) proteins [23]. RNA m6A modification is abundant in the near stop codons and 3′ untranslated terminal regions (3′UTRs), which affect the expression of a transcriptome [24]. Increasing evidence shows that the diversity of m6A methylation is associated with multiple biological functions in mammals, such as mRNA splicing procession [25], mRNA stability [26], protein translation efficiency [27], and cell differentiation [28] (Figure 2(a)). Along with the recent breakthrough findings of two mammalian RNA demethylases, FTO (the fat mass and obesity-associated protein) [29] and ALKBH5 (alkylation repair homolog protein 5) [30], the dynamic and reversible modification of m6A methylation in physiological processes has begun to be appreciated [31, 32]. Disturbing m6A RNA methylation might be associated with cancer metastasis. Abnormal expression of the writer, eraser, and reader proteins of m6A was strikingly linked with cancer advance. For instance, in vitro and in vivo experiments have shown that METTL14 (methyltransferase-like 14) inhibition significantly promoted tumor metastasis by modulating the primary microRNA 126 process in an m6A-dependent manner [33]. And loss of function of METTL3 decreased protein abundance for several m6A-containing mRNAs but had minimal impact on mRNA levels. METTL3 expression was upregulated in lung adenocarcinoma, and using its knockdown and overexpression studies demonstrated that METTL3 promoted invasion of human lung cancer cells [34]. Furthermore, expression of metastasis-associated genes shaped by m6A methylation was one of the key factors for cancer metastasis. As an example, TRIM7 expression was influenced by m6A modification and regulated osteosarcoma cell migration and invasion [35]. With the application of more and more sensitive detection techniques, researchers will discover the distribution of m6A methylation in transcriptomics and their associated fundamental properties in cancer [36]. Epigenetic modification of RNA is a rapidly developing field in biology, and the knowledge of its modification mainly focuses on m6A, which gives us a promising future therapeutic strategy in cancer. Clarifying the role of m6A modification in genes and their classical mechanisms involved in tumor metastasis will probably be the focus of future research.

#### 1.1.3. Histone Methylation

Histone modification, as a key characteristic of epigenetic regulation, has an important role in cancer progression. A diverse set of identified histone modifications known in cancer are deregulated, such as methylation, acetylation, phosphorylation, sumoylation, adenosine diphosphate (ADP) ribosylation, deimination, and crotonylation [37]. Misregulation of these modifications can lead to inappropriate activation of oncogenes or tumor suppressors [5]. Currently, methylation modification of histone has been considered a potential target for cancer treatment, and clinical trials have shown promising results [38]. It has been well documented that how histone methylation was modified relative to writers, erasers, and readers dynamically regulated methylation in association with cancer [12]. It is widely accepted that dysregulation of histone methylation may be a driver in diverse types of cancer. Here, we focus on the latest reports on histone methylation in cancer metastasis. There are at least four mechanisms that explain methylation's role in metastasis (Figure 2(b)). Firstly, histone modification affects the recruitment of the transcriptional factor, which is linked with dysregulated expression of metastasis-linked genes [39]. It was reported that H3K9 histone methyltransferase G9a enhanced the level of H3K9 dimethylation, which contributed to the recruitment of the transcription cofactors HP1, DNMT1, and HDAC1 into the promoter region of the cell adhesion molecule Ep-CAM, thus inhibiting the expression of Ep-CAM and promoting the invasion and metastasis of lung cancer [40]. Secondly, histone methyltransferase and demethylase coordinate to regulate the expression of prometastatic genes [41, 42]. Thirdly, histone modification takes part in modulating the tumor microenvironment through molecular signaling pathways to promote cancer progression and metastasis [43]. Fourthly, the altered microenvironment conditions in cancer initiate respondent sensor reactions by controlling the histone methyltransferase activity, leading to dysregulated expression of metastasis genes. For instance, under the hypoxia condition of ovarian cancer, FIH, as an oxygen sensor, drove the expression of metastasis-related genes through histone lysine methyltransferases G9a and GLP [44]. Multiple known mechanisms can regulate the expression of metastasis-related genes in a synergistic or interdependent manner, and they can be combined with sensor reaction in the abnormal microenvironment to participate in tumor metastasis [45]. In summary, histone modification provides a critical regulatory role in cancer metastasis-linked gene transcription and microenvironment-induced cancer progression. As a form of epigenetic therapy, some inhibitors of histone modification have shown efficacy in preclinical trials. Inhibitors of EZH2 (enhancer of zeste homolog 2, H3K27 methyltransferase [46]), DOT1L (H3K79 methyltransferase [47]), PRMT1 (arginine N-methyltransferases [48]), and PRMT5 have now entered clinical trials [12]. The data from these studies will benefit a better understanding of the role of histone modifications in cancer progression and give new hope for cancer treatment.

### 1.2. Alternative Splicing as a Regulator of Cancer Metastasis

**Figure 3:**
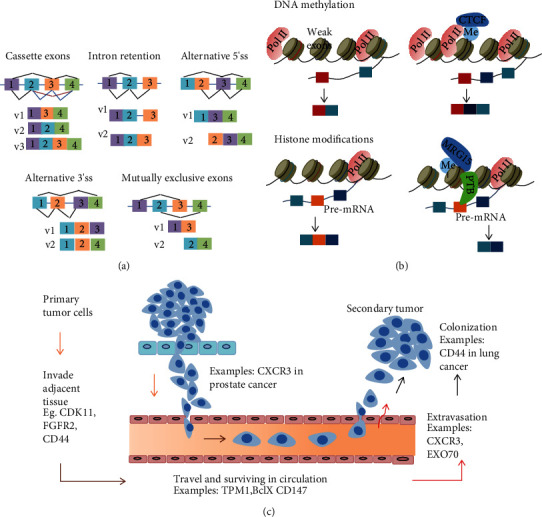
Schematic diagram of an alternative splicing process and its relative function in metastasis. (a) The five different alternative splicing types. (b) DNA methylation or histone methylation influenced the production of alternative splicing. (c) Alternative splicing is involved in every step of metastasis, from primary tumor cells invading adjacent tissues to moving to new sites, surviving in the circulation, and adapting to distant tissues to form secondary tumor (colonization).

Alternative splicing acts on the precursor messenger RNA (pre-mRNA) of one gene to generate diverse isoforms of RNA and protein [49]. In humans, up to 94% of genes undergo alternative splicing [50]. These different isoforms of mRNA can affect their own stability, localization, or translation [51]. And relevant protein isoforms may have related, distinct, or even opposing functions that vary from tissue to tissue [50]. No matter whether it is in normal tissues or in cancer progression, there are at least five main alternative splicing patterns (Figure 3(a)): cassette exons, intron retention, alternative 5′ splice sites, alternative 3′ splice sites, and mutually exclusive exons [52]. Histone modifications or DNA methylation has been reported to have a direct effect on alternative splicing [53] (Figure 3(b)). For example, several studies showed significant aggregation of the H3K36me3 (histone H3 lysine 36 trimethyl) signals on exons, and these histone signals might be associated with lower levels in alternatively spliced exons compared to constitutive exons [54]. In addition, with technological advances in single-cell sequencing, researchers have used scM&T-seq (single-cell methylation and transcriptome sequencing) [55] to discover the correlation and variation between transcription and methylation. It also has been found that local DNA methylation profiles influence splicing variation across cells. And DNA methylation information might accurately predict different splicing patterns of individual cassette exons during cell differentiation [56]. DNA methylation promoted the exclusion of weak upstream exons by inhibiting DNA-binding protein function in genes, such as CCCTC-binding factor- (CTCF-) recognized CD45 exon 5 inhibited by DNA methylation 5mC, which mediated local RNA polymerase II pausing and linked to alternative splicing [57]. Alternative splicing is not only an important mechanism for cell development, differentiation, and regulation of tissue-specific function [51], but it has also been found in a variety of pathological processes, including tumorigenesis and cancer metastasis [58]. And alternative splicing has been used as a relevant therapeutic target for cancer treatment [59]. Alternative splicing is involved in most stages of metastasis, including EMT (epithelial-mesenchymal transition) [60], stemness feature [60], invasion [61], surviving in circulation [62], extravasation [61], and colonization in new sites [63] (Figure 3(c)). Here, epithelial-restricted splicing regulator (ESRP) is a salient example of how alternative splicing promotes cancer metastasis. Inhibition of the expression of ESRP is enough to make some changes in morphology in epithelial cells and contribution to EMT [69]. ESRP also mediates the splicing event of EMT-related genes in their pre-mRNA to produce different isoforms, such as p120 and NUMB [70, 71]. Although an enormous work has been done on the roles of alternatively splicing in cancer metastasis, it is still important to identify the key molecules and epigenetic modifications that control the abnormal subtypes of proteins, which contribute to disease progression in certain types of cancer.

### 1.3. Noncoding RNAs That Play an Increasingly Important Role in Tumor Metastasis

The effects of noncoding RNAs on physiological and pathological processes are increasingly becoming clear. Now we know that noncoding RNAs interact with DNA, protein, or RNA to execute their function [71]. For example, noncoding RNAs have been documented to change protein contents by regulating gene expression at the transcriptional level [72] or by altering mRNA translational efficiency [73]. Noncoding RNAs can also change the location of proteins by turning their signaling pathways [74], or act as working partners of proteins [75]. The discovery of these novel noncoding RNAs has sparked a new round of exploration and further research into cancer. Here, we present recent advances and explore how noncoding RNAs are involved in cancer metastasis. The well-known noncoding RNAs, including tRNAs and rRNAs, are in abundance and have well-defined regulatory roles in translation. Novel classes of noncoding RNAs emerged more than two decades ago, for example, mircroRNAs (miRNAs), circular RNAs (circRNAs), and long noncoding RNAs (lncRNAs). Accumulating evidence suggests that noncoding RNAs have essential roles in regulating gene expression and cancer metastasis.

#### 1.3.1. tRNA-Derived ncRNAs

**Figure 4:**
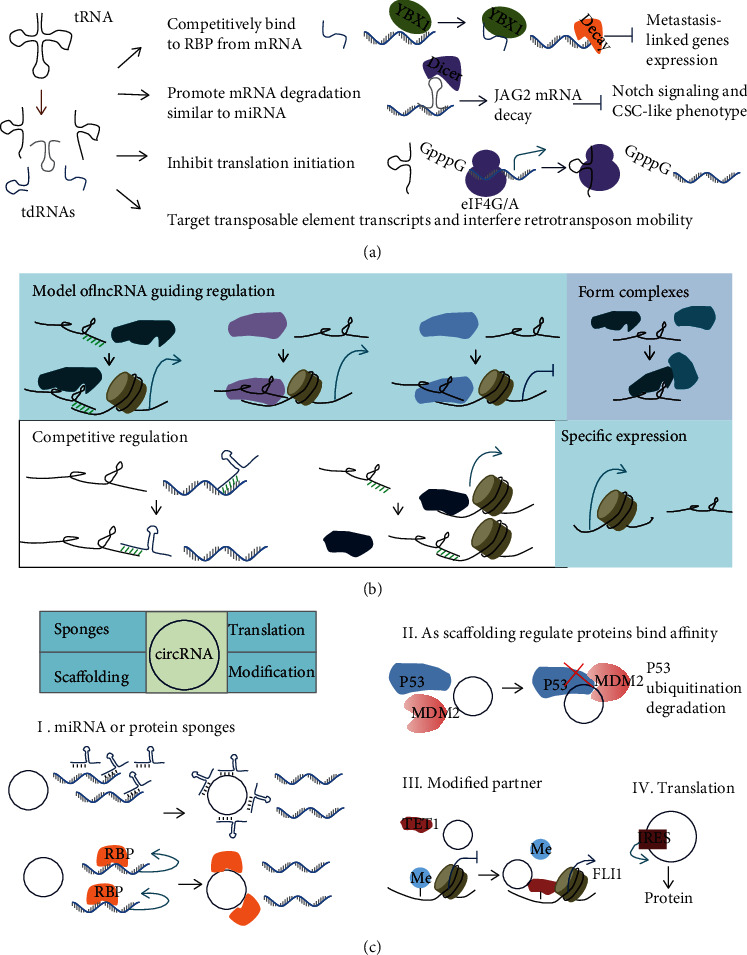
Diverse mechanisms of noncoding RNAs in metastasis. (a) tRNA-derived ncRNAs (tdRNAs) inhibited the mRNA expression of metastasis-linked genes by competing with its work-partner RBP, promoting mRNA degradation, inhibiting its translation, or interfering with retrotransposon mobility. (b) Schematic shows long noncoding RNAs (lncRNAs) working in cancer progression through a variety of molecular mechanisms. lncRNAs guided proteins, such as transcription factors, to specific sites and further affected the expression of downstream genes by complementary base pairing or complex formation. lncRNAs also competed with miRNAs or proteins to bind to mRNA, thereby affecting its stability or translation initiation. The specific expression of lncRNAs were also found in metastasis. (c) circRNAs worked in metastasis. circRNAs acted as competing endogenous RNAs (ceRNA) of proteins or miRNAs, as modified partners, or disturbed metastasis-linked gene transcription or translation.

tRNA-derived ncRNAs (tdRNAs) have been discovered in a variety of organisms and species [76]. tdRNAs, as a novel class of small noncoding RNAs, are generated from specific cleavages of mature or precursor tRNAs. According to the cleavage position, tsRNAs can be currently categorized into at least three types [77]. tRNA-derived small RNAs (tsRNAs) and tRNA-derived fragments (tRFs or tDRs) are generated during the maturation of tRNA precursors and maturation sequences, respectively. The third type is the tRNA halve (tiRNA), which is generated from the production of mature tRNAs that undergo cleavage in the anticodon loops. tdRNAs are believed to play important roles in diverse aspects of biological processes, for instance, cell proliferation [78], regulation of gene expression [79], stress response [80], control of retrotransposon [81], and tumor suppression [82]. tdRNAs also have functions in cancer metastasis (Figure 4(a)). Here, we summarize the known mechanisms of tdRNAs in promoting the cancer process. First of all, tdRNAs competed with the mRNAs of metastasis-linked genes at binding sites for functional proteins, such as RNA-binding proteins. For example, endogenous tdRNAs, like tRFs, tRF^Asp^, tRF^Gly^, tRF^Glu^, and tRF^Tyr^, competed with multiple metastasis-relative transcriptions for their binding sites in RNA-binding protein YBX1. The competitive binding to YBX1 resulted in the destabilization of metastasis-relative transcriptions that suppressed cancer cell invasion and metastasis [82]. In addition, tdRNAs are functionally similar to miRNAs in that they downregulate targeted genes and interfere with their related biological processes. For instance, tRF/miR-1280, derived from both tRNA^Leu^ and pre-microRNA, was significantly decreased in human colorectal cancer (CRC) tissues. And tRF/miR-1280 inhibited the Notch signaling pathway and relative metastatic features by directly downregulating JAG2 expression, which leads to the cancer stem cell-like phenotype [83]. Furthermore, tdRNAs were specifically generated by cleavage within tRNA anticodon loops using ribonuclease angiogenin under stress-activated conditions. And the incision production of 5′-tiRNAs, not 3′-tiRNAs, inhibited translation and protein synthesis by competing to displace eIF4G/eIF4A from mRNA-binding sites [84]. Over the past 40 years, the understanding of tdRNAs has undergone dramatic changes, from meaningless degradation products of tRNA to key players in orchestrating gene regulation in physiological or pathological processes. However, the multifaceted regulatory potential of tdRNAs in cancer still requires deeper validation. It is a challenge to find out key tdRNAs as biomarkers of cancer processes and new approaches to targeted tdRNAs for cancer diagnosis and treatment.

#### 1.3.2. lncRNAs

lncRNAs are identified by their transcription length of more than 200 nucleotides, without or with partly protein-coding potential, and many of them are uniquely expressed in tissues and cells. lncRNAs influence a number of cellular functions in pathological and physiological processes; for example, lncRNAs have a variety of functions in tumorigenesis and metastasis by regulating the expression of targeted genes [85]. It has been reviewed that lncRNAs are involved in cancer phenotypes, like proliferation and invasion, through interactions with DNA, protein, or RNA [70, 86]. Because of lack of common characteristics, the existing lncRNAs can be classified according to their sequence features, biochemical pathways, subcellular location, or functions [87]. And the sequences of lncRNAs are poorly conserved; however, their action modes are similar. Here, we segregated their action models into four categories in cancer metastasis (Figure 4(b)). First, lncRNAs act as guides for downstream molecules to a special location. For example, upregulation of lncRNA HOTAIR indicated an increased risk of tumor invasion and metastasis in primary breast tumors. And the molecular mechanism was HOTAIR dysregulated downstream metastasis-linked gene expression by guiding Polycomb Repressive Complex 2 (PRC2) to histone H3 altering lysine 27 methylation [88]. Furthermore, lncRNA may be competitive with other molecules. For instance, lncRNA-ATB competitively binds with the miR-200 family from their targeted ZEB1 and ZEB2 gene, which indirectly contributes to the expression of the oncogene ZEB1/2 and promotes EMT and invasion in hepatocellular carcinoma [89]. Third, lncRNAs act as scaffolds to facilitate the molecules in executing their functions. lncRNA-mPvt1, as an example, is bound to NOP2 protein and enhances its stability, which is beneficial to cell proliferation, cell cycling, and the acquisition of stem cell-like properties in hepatocellular carcinoma (HCC) cells [90]. Lastly, lncRNAs may be specifically expressed in tissues or cells [91]. With more and more evidence showing that lncRNAs are emerging as important actors involved in cancer metastasis, a better understanding of the regulation and regulatory mechanism of lncRNA will shed light on the hope for novel clinical treatment.

#### 1.3.3. miRNAs

MicroRNAs (miRNAs) are noncoding RNAs with a length of about 18~22 nucleotides that regulate gene expression by binding to the mRNA 3′UTR of targeted genes, leading to translational repression or mRNA decay [92]. It has been well studied how miRNAs are involved in the initiation, progression, and inhibition of cancer [93]. And miRNAs have been shown potential roles in the diagnosis, prognosis, and treatment in cancer clinical applications [94]. About 30% of human genes are regulated by miRNAs, and miRNA expression profiles might be associated with cancer types and their stages [95]. In general, miRNA expression is always downregulated in cancer compared with normal tissues [95]. Since miRNAs mediating metastasis were reported in 2007, various mechanisms about how miRNAs work in metastasis have been found [3]. Now, a growing body of evidence demonstrated that miRNA mediated metastasis gene expression, cancer stem cell-like phenotype, epithelial-to-mesenchymal transition (EMT), invasion, and migration, remodeling the tumor tissue microenvironment by exosomes [96]. Here, we selected 3 examples for each of these five aspects, as shown in Table 2. The key miRNAs related to metastasis specifically in cancer could be used as biomarkers for diagnosis and therapeutic target.

#### 1.3.4. circRNAs

Since the discovery of the first circRNA in 1976 [115], circRNAs have gone from being considered as “junk” generated by nonsense splicing events to a large class of noncoding RNAs exerting important biological functions in physiological or pathological processes [116]. circRNAs lack poly(A) tails and 5′ termini capping, and most of them come from known protein-coding genes by a back-splicing event [117]. And their expression have tissue- and cell-specific patterns [118, 119]. Studies have explored circRNAs involved in cancer with a steadily increasing pace, and there are at least four potential ways to elucidate the mechanisms of circRNAs in cancer (Figure 4(c)). Firstly, circRNAs act as miRNAs or protein sponges that indirectly regulate the functions of their downstream target genes. For example, circTP63 function as a competitive endogenous RNA (ceRNA) of miR-873-3p, thereby inhibiting miR-873-3p function on FOXM1, which finally leads to upregulating FOXM1 expression and cell cycle progression in lung squamous cell carcinoma [120]. In another example, HuR is a well-studied RNA-binding protein (RBP) regulating a range of RNA expression. The binding site of HuR and PABPN1 mRNA was blocked by circPABPN1, and the competition decreased the translation of PABPN1 [121]. Secondly, circRNAs work as protein scaffolds to regulate binding affinity. circ-Foxo3, as a case in point, promoted MDM2-induced p53 ubiquitination but prevented Foxo3 ubiquitination and affected subsequent degradation [122]. In addition, circRNAs recruit proteins to specific genomic locations and cooperate with epigenetic modifications to regulate the expression of downstream genes; for example, circular RNA FECR1 recruited TET1, a DNA demethylase, to the FLI1 promoter and activated FLI1 expression [123]. Furthermore, a number of recent studies have shown that circRNA own an open reading frame (ORF), and an internal ribosome entry site (IRES) can be translated into functional proteins [124]. Lastly, a subset of circRNAs that is dominantly localized in the nucleus may interact with the Pol II transcription complex to enhance transcription of their parent coding genes [125]. circRNAs used in translational and precision medicine researches has become a hotspot.

## 2. Conclusions and Future Perspective

In tumor progression, finding the key epigenetic molecules that control metastasis is of great clinical significance and will provide a new way to search for effective therapeutic drugs and molecular markers for diagnosis. Here, we present the available evidence from four parts on how epigenetics plays a role in cancer metastasis. First, DNA methylation or histone methylation, either alone or in combination with other epigenetic molecules (such as noncoding RNA), regulated the expression of metastasis-linked genes at the transcriptional level. Second, RNA methylation also took part in translation initiation, translation efficiency, and mRNA stability of metastasis-linked genes. Third, alternative splicing increased the diversity of mRNA and noncoding RNA that played important roles in tumor metastasis, and these processes were partly influenced by methylation modification of DNA or RNA. Fourth, noncoding RNAs were almost involved in the whole process of metastasis-linked genes from transcription initiation to protein production to biological function. It has been reported that some early clinical trials targeted epigenetics, for instance, by designing drugs that specifically target DNA methylation or histone methylation for cancer therapeutics (see the review in [12]). Epigenetic drugs' combination strategies, such as DNMT inhibitors combined with HDAC inhibitors for the treatment of myelodysplastic syndrome and acute myeloid leukemia, have also been investigated in clinical trials. [126]. While many researches have explored the role of epigenetics as a biomarker and have addressed key pathways driving disease processes, there are still some major challenges that exist in their application in broader clinical medicine. For example, how to ensure the accuracy and repeatability of the measurements, how to distinguish transient changes in disease from the true biomarkers, and how to translate the results into the precise medication for the individual to make sure maximum benefit for patients [127]. Epigenetic therapies present opportunities and challenges, and further research is still needed to translate these relatively significant findings into clinical applications.

## Figures and Tables

**Table 1:** Examples of DNA methylation with cancer metastasis.

Gene name	DNA methylation location/level	Cancer types	Samples	Refs
POPDC1, POP1	Promoter, hypermethylation	Colorectal carcinoma (CRC), adenomatous polyps	Human tissues, CRC cell lines, athymic mice	[64]

Akr1b1, Hoxb4, Rasgrf2, Rassf1, Hist1h3c, Tm6sf1	High cumulative methylation index	Breast cancer	Serum, 182 women	[20]

IRX1	Hypomethylation	Osteosarcoma	2 cell lines, serum from 67 primary osteosarcoma	[21]

Genome-scale analyses of DNA methylation	High consistency of hypermethylation across metastases	Lethal metastatic prostate cancer	Tissue, 71 specimens from each of 13 subjects	[65]

2481 differentially methylated regions	CpG island, flanking regions, and CpG sparse promoters	Prostate cancer	17 tissues, 6 cell lines	[66]

HOP homeobox HOPX	Promoter, hypermethylation	Nasopharyngeal carcinoma	443 formalin-fixed paraffin-embedded (FFPE) NPC tissues, 5 human NPC cell lines	[67]

47 genes (RDBP), 48 CpGs significant association	Hypermethylation	Pheochromocytoma, paraganglioma	310 tumors were obtained from patients, 67 with metastases	[68]

**Table 2:** List of examples of miRNA-mediated pathways in cancer metastasis.

Relative phenotypes	Pathways	Cancer types	Refs
Metastasis gene expression	mir-9-E-cadherin	Breast cancer	[97]
	miR-1269a-Smad7 and HOXD10	Colorectal cancer	[98]
	miR-122-ADAM17	Hepatocellular carcinoma	[99]

Cancer stem cell-like phenotype	miR-199a-FOXP2	Breast cancer	[100]
	miR-34a-CD44	Prostate cancer	[101]
	miR-23a-metastasis suppressor 1 (MTSS1)	Colorectal cancer	[102]

EMT	miR-30a-Snai1	Non-small-cell lung cancer	[103]
	miR-200 family/miR-205-ZEB1/SIP1	Breast cancer	[104]
	miR-1296-SRPK1	Hepatocellular carcinoma	[105]

Invasion and migration	miR-21-programmed cell death 4/maspin	Breast cancer	[106]
	miR-132-ZEB2	Colorectal cancer	[107]
	miR-940-migration and invasion enhancer 1	Prostate cancer	[108]

Remodeling microenvironment by exosomal miRNA	miR-10b-HOXD10 and KLF4	Breast cancer	[109]
	miR-17, miR-19a, miR-19b, miR-20a, and miR-92-PTEN	Brain metastasis	[110]
	miR-103-VE-cadherin, p120-catenin, and zonula occludens 1	Hepatocellular carcinoma	[111]

Metastasis biomarker	miR-203	Colorectal cancer	[112]
	miR-1246 and miR-1290	Non-small-cell lung cancer	[113]
	miR-200b	Breast cancer	[114]
